# Supplementary material for: Nanoparticle-based targeting of microglia improves the neural regeneration enhancing effects of immunosuppression in the zebrafish retina
Source: Commun Biol. 2023 May 18;6:534. doi: 10.1038/s42003-023-04898-9 (PMC10193316; doi:10.1038/s42003-023-04898-9)
Supplement: Supplementary file 2 — Supplementary Information [file 42003_2023_4898_MOESM2_ESM.pdf]

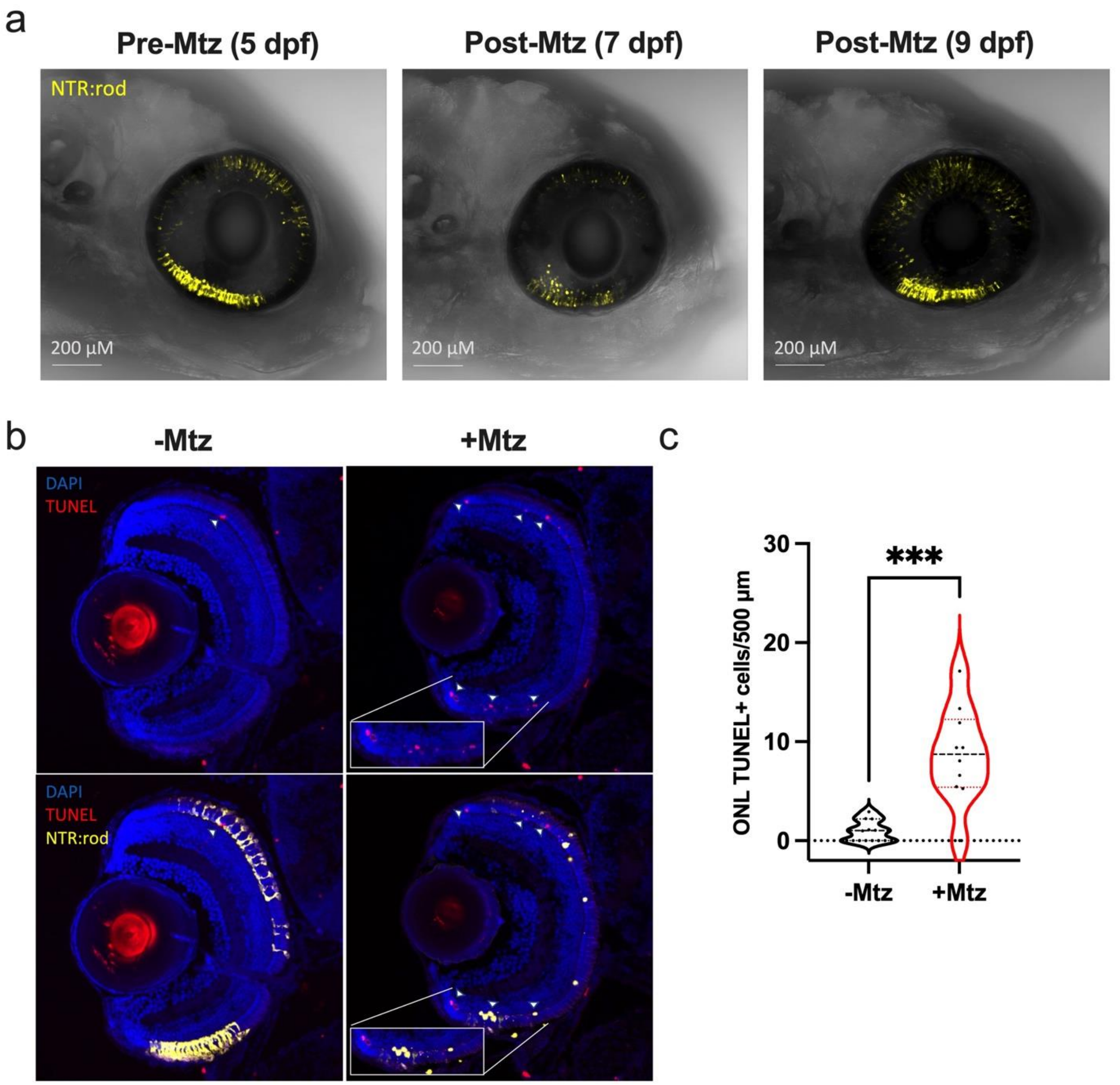

Supplementary Figure 1. NTR-based model of inducible rod photoreceptor death.

a 3D-projection confocal time series images of a transgenic NTR:rod larva taken prior to Mtz-mediated rod cell ablation (Pre-Mtz, 5 dpf, NTR-YFP expressing rod cells are labeled yellow), two days after removal of Mtz, to assess rod cell loss (Post-Mtz, 7dpf), and following a further 48h of recovery to assess rod cell regeneration (Post-Mtz, 9 dpf). **b** Representative images of retinal sections from NTR:rod larvae treated ±Mtz and stained for DAPI (blue) and TUNEL (red). Control non-ablated larvae were not exposed to Mtz (-Mtz, left panels), ablated larvae were treated with 10 mM Mtz from 5-6 dpf (+Mtz, right panels), white arrowheads indicate TUNEL$^+$ cells (red). **c** Quantification of the average number of TUNEL$^+$ cells in the outer nuclear layer (ONL) of -Mtz and +Mtz larvae, n=10 for each group (***p≤0.001).

Supplementary Figure 2. Dex does not protect rod photoreceptors from NTR-mediated cell death.

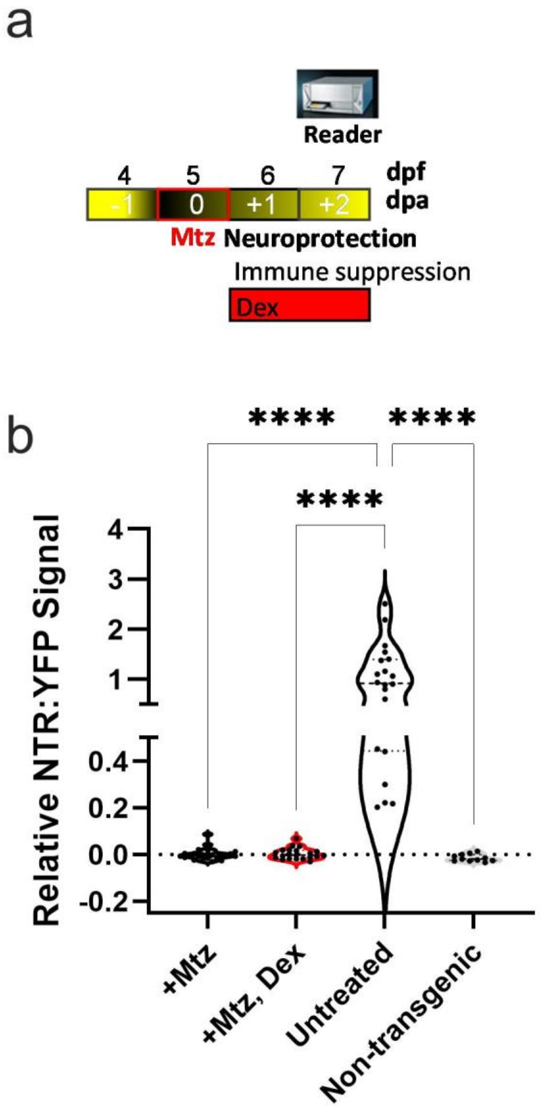

a Assay schematic, at 5 dpf NTR:rod larvae were split into untreated and +Mtz groups with half of the +Mtz group being treated with 2.5 µM Dex 4 h prior to exposure to 10 mM Mtz for 24 h (5-6 dpf). NTR-YFP fluorescence was quantified at 7 dpf (non-transgenic larvae were used to establish signal cutoff). **b** YFP signals plotted relative to the untreated controls, n= 24, 21, 20 and 11 from left to right (****p≤0.0001, all other comparisons were not statistically significant).

Supplementary Figure 3. Microglia reactivity is not altered by Mtz treatment alone.

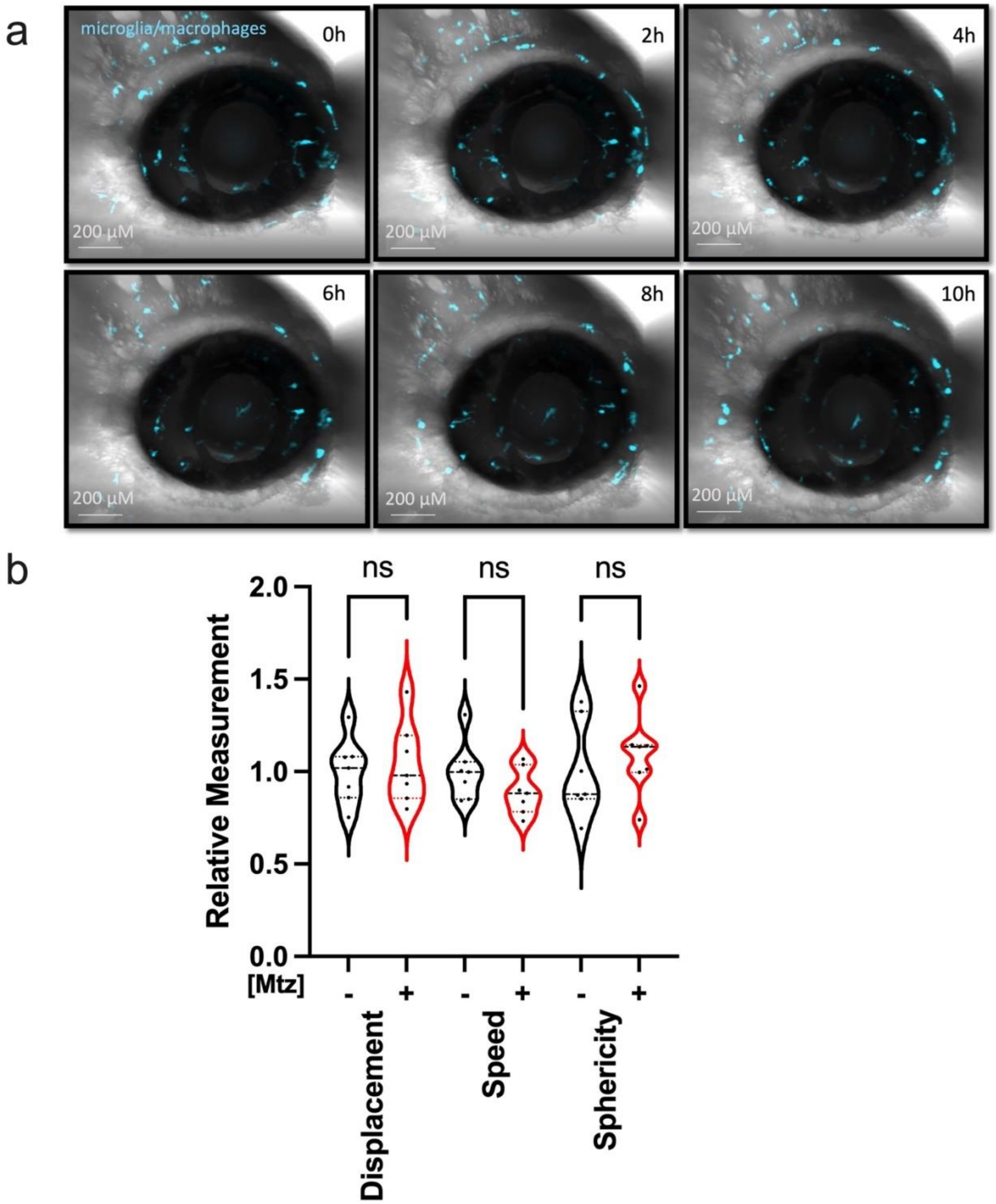

Supplementary Figure 3. Microglia reactivity is not altered by Mtz treatment alone.

a Representative 3D-projection confocal images from a 12h time-lapse series of a non-ablated control larva with labeled microglia (cyan) but without the NTR-YFP transgene. Imaging began immediately after a 10mM Mtz treatment to assess Mtz effects on microglia behavior in the absence of rod cell loss (see Supplementary Video 5 for corresponding time-lapse sequence). **b** Imaris-based quantification of microglia displacement, speed and relative sphericity compared between untreated (-Mtz) and +Mtz larvae over 4 h of time-lapse imaging initiated at 4 h post-Mtz exposure (n= 7 larva per condition; ns, indicates no statistically significant differences).

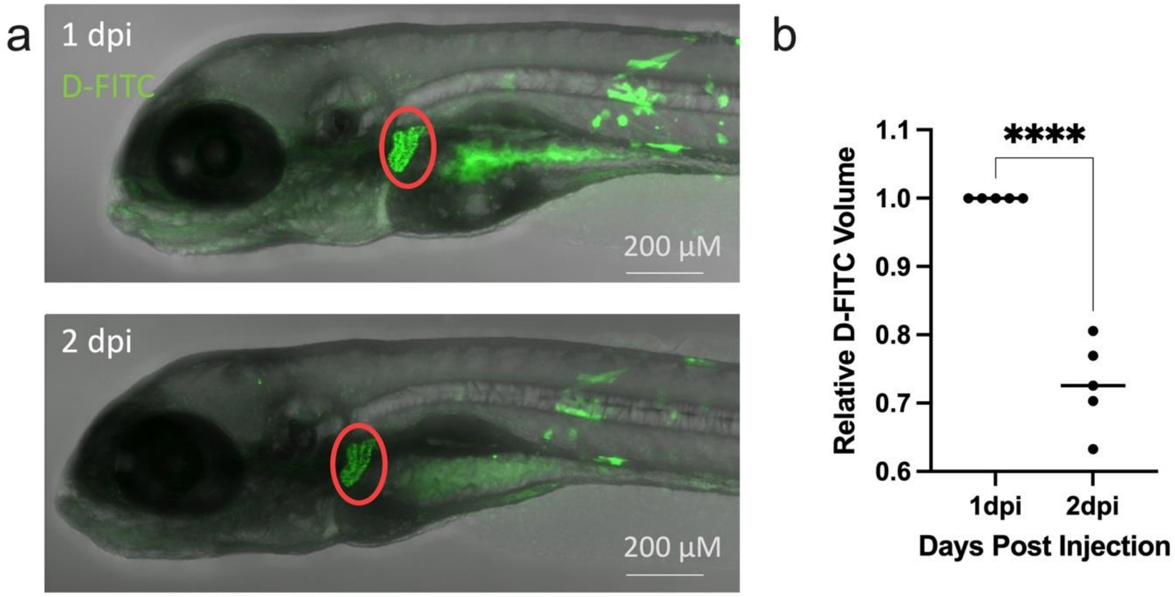

Supplementary Figure 4. Dendrimer clearance in the absence of rod cell loss.

a Representative 3D-projection confocal time series images of larva with FITC-conjugated dendrimers (D-FITC, green) injected into the pericardium at 5 dpf, and imaged at 6 and 7 dpf (1 and 2 dpi, days post injection). **b** Imaris-based volumetric quantification of D-FITC in the kidney (red circles) at 1 and 2 dpi (n=5 fish, 2 dpi signals were normalized to 1 dpi signals per each individual fish, ****p≤0.0001).

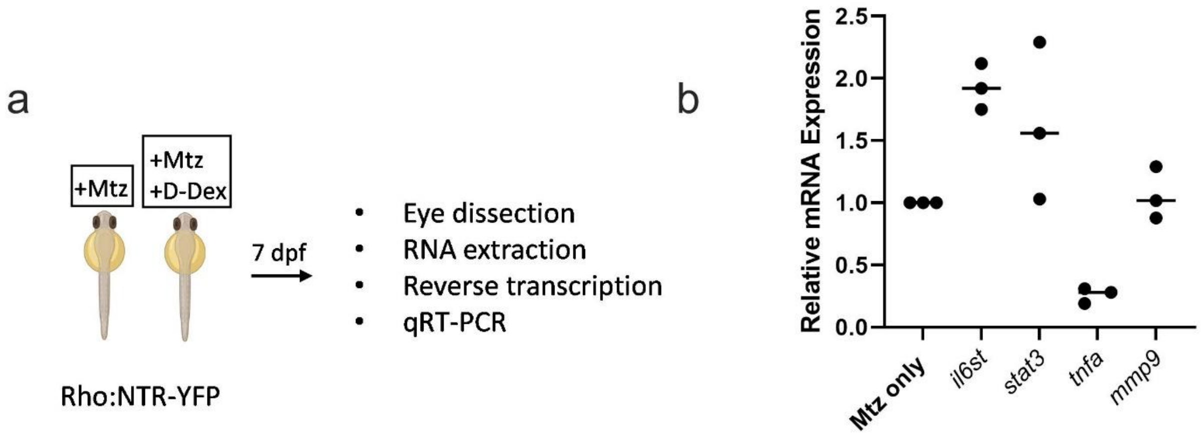

Supplementary Figure 5. Quantitative real-time PCR for select inflammatory factors.

a Assay schematic for qRT-PCR: NTR:rod larvae treated with 10 mM Mtz from 5-6 dpf with half then being injected with D-Dex from 6-7 dpf. At 7 dpf eyes were dissected and processed for qRT-PCR. **b** Normalized expression levels of *il6st*, *stat3*, *tnfa* and *mmp9* for +Mtz, +D-Dex larvae across 3 biological replicates (sample size was 30 larval eyes per replicate). Fish icons in panel (a) were produced with permissions from Biorender.
