# Supplementary material for: Nanoparticle-based targeting of microglia improves the neural regeneration enhancing effects of immunosuppression in the zebrafish retina
Source: Commun Biol. 2023 May 18;6:534. doi: 10.1038/s42003-023-04898-9 (PMC10193316; doi:10.1038/s42003-023-04898-9)
Supplement: Supplementary file 3 — Description of Additional Supplementary Files [file 42003_2023_4898_MOESM3_ESM.pdf]

Description of Additional Supplementary Files

File name: Supplementary Data 1

Description: All hit genes identified in bulk RNA-seq as well as gRNAs ordered to target genes with CRISPR.

File name: Supplementary Data 2

Description: All source data for plots.

File name: Supplementary Movie 1

Description: Source behind image clips in Figure 1a and quantification in 1e-g.

File name: Supplementary Movie 2

Description: Source behind image clips in Figure 1b and quantification in 1e-g.

File name: Supplementary Movie 3

Description: Source behind image clips in Figure 1c and quantification in 1e-g.

File name: Supplementary Movie 4

Description: Source behind image clips in Figure 1d and quantification in 1e-g.

File name: Supplementary Movie 5

Description: Source behind image clips and quantification in Supp figure 3.

File name: Supplementary Movie 6

Description: Source behind image clips and quantification in Figure 3 for Mtz ablation.
