# Supplementary material for: Nanoparticle-based targeting of microglia improves the neural regeneration enhancing effects of immunosuppression in the zebrafish retina
Source: Commun Biol. 2023 May 18;6:534. doi: 10.1038/s42003-023-04898-9 (PMC10193316; doi:10.1038/s42003-023-04898-9)
Supplement: Supplementary file 12 — Reporting Summary [file 42003_2023_4898_MOESM12_ESM.pdf]

nature portfolio

Corresponding author(s): Dr. Jeff Mumm, Dr. Eric Betzig, Dr. Kannan Rangaramanujam

Last updated by author(s): 04/27/2023

Reporting Summary

Nature Portfolio wishes to improve the reproducibility of the work that we publish. This form provides structure for consistency and transparency in reporting. For further information on Nature Portfolio policies, see our Editorial Policies and the Editorial Policy Checklist.

Statistics

For all statistical analyses, confirm that the following items are present in the figure legend, table legend, main text, or Methods section.

n/a	Confirmed	
☐	☒	The exact sample size (*n*) for each experimental group/condition, given as a discrete number and unit of measurement
☐	☒	A statement on whether measurements were taken from distinct samples or whether the same sample was measured repeatedly
☐	☒	The statistical test(s) used AND whether they are one- or two-sided<br>*Only common tests should be described solely by name; describe more complex techniques in the Methods section.*
☒	☐	A description of all covariates tested
☐	☒	A description of any assumptions or corrections, such as tests of normality and adjustment for multiple comparisons
☐	☒	A full description of the statistical parameters including central tendency (e.g. means) or other basic estimates (e.g. regression coefficient) AND variation (e.g. standard deviation) or associated estimates of uncertainty (e.g. confidence intervals)
☐	☒	For null hypothesis testing, the test statistic (e.g. *F*, *t*, *r*) with confidence intervals, effect sizes, degrees of freedom and *P* value noted<br>*Give P values as exact values whenever suitable.*
☒	☐	For Bayesian analysis, information on the choice of priors and Markov chain Monte Carlo settings
☒	☐	For hierarchical and complex designs, identification of the appropriate level for tests and full reporting of outcomes
☒	☐	Estimates of effect sizes (e.g. Cohen's *d*, Pearson's *r*), indicating how they were calculated

Our web collection on statistics for biologists contains articles on many of the points above.

Software and code

Policy information about availability of computer code

Data collection

No software used in data collection

Data analysis

Raw sequencing data (in the form of fasta.gz files) was downloaded and unzipped and then terminal function cutadapt was used to remove Nextera sequencing adapters (specifically the sequence CTGTCTCTTATA was trimmed). Samples were then read into fastqc for quality control to ensure proper and similarly sized libraries, complete removal of adapters and high sequence quality scores throughout. Sequencing for each sample occurred over two lanes and thus read files were combined using the "cat" terminal function. Read files were then mapped to the most recent Ensembl reference genome for Danio rerio GRCz11/danRer11 using kallisto. Percentage of uniquely mapped reads was identified by fastqc and ranged from 69-75% for the 15 samples.

To identify differentially expressed genes (DEGs) between conditions, a matrix file containing all Ensembl transcript ID's and 12 columns, one for each sample, were read into the edgeR Bioconductor package (version 3.34.1) in R/R Studio (versions 4.0.3 and 1.4.1103, respectively) 66. Briefly, a model matrix was designed to aggregate data between 3 biological replicates for each condition. Further analysis of the variance between samples revealed low quality data for one replicate of Mtz only RNA compared to the other groups and thus this group was removed. Next, all possible pairwise comparisons between the 4 samples were completed using the glmLRT method. Data was then exported as .csv files. Prior to identifying hit DEGs, control sample comparisons were used to filter out transcripts for comparisons of interest. Finally, remaining transcripts were sorted to identify hit DEGs that demonstrated at least a log fold change in expression of 1 (an expression fold change of 2) in either direction and with a false discover rate (FDR) <0.10.

For manuscripts utilizing custom algorithms or software that are central to the research but not yet described in published literature, software must be made available to editors and reviewers. We strongly encourage code deposition in a community repository (e.g. GitHub). See the Nature Portfolio guidelines for submitting code & software for further information.

Data

Policy information about availability of data

All manuscripts must include a data availability statement. This statement should provide the following information, where applicable:

- Accession codes, unique identifiers, or web links for publicly available datasets
- A description of any restrictions on data availability
- For clinical datasets or third party data, please ensure that the statement adheres to our policy

Bulk RNA-sequencing data availability
Sequencing data that support the findings of this study have been deposited in GEO with the accession code GSE216060.

Human research participants

Policy information about studies involving human research participants and Sex and Gender in Research.

Reporting on sex and gender	*Use the terms sex (biological attribute) and gender (shaped by social and cultural circumstances) carefully in order to avoid confusing both terms. Indicate if findings apply to only one sex or gender; describe whether sex and gender were considered in study design whether sex and/or gender was determined based on self-reporting or assigned and methods used. Provide in the source data disaggregated sex and gender data where this information has been collected, and consent has been obtained for sharing of individual-level data; provide overall numbers in this Reporting Summary. Please state if this information has not been collected. Report sex- and gender-based analyses where performed, justify reasons for lack of sex- and gender-based analysis.*
Population characteristics	*Describe the covariate-relevant population characteristics of the human research participants (e.g. age, genotypic information, past and current diagnosis and treatment categories). If you filled out the behavioural & social sciences study design questions and have nothing to add here, write "See above."*
Recruitment	*Describe how participants were recruited. Outline any potential self-selection bias or other biases that may be present and how these are likely to impact results.*
Ethics oversight	*Identify the organization(s) that approved the study protocol.*

Note that full information on the approval of the study protocol must also be provided in the manuscript.

Field-specific reporting

Please select the one below that is the best fit for your research. If you are not sure, read the appropriate sections before making your selection.

☒ Life sciences ☐ Behavioural & social sciences ☐ Ecological, evolutionary & environmental sciences

For a reference copy of the document with all sections, see nature.com/documents/nr-reporting-summary-flat.pdf

Life sciences study design

All studies must disclose on these points even when the disclosure is negative.

Sample size	Sample size was always determined based on the number of individual larvae tested in each in vivo experiment or the number of distinct quantified retinal sections for histology
Data exclusions	Bulk RNA Seq data: Further analysis of the variance between samples revealed low quality data for one replicate of Mtz only RNA compared to the other groups and thus this group was removed.
Replication	All in vivo experiments were repeated multiple times. For bulk RNA sequencing experiments multiple groups for each condition were individually treated and then sequenced in the same run.
Randomization	For all experiments following identification of transgenic fish, they were then randomly split into each experimental group prior to the experiment
Blinding	In these experiments blinding was not conducted or necessary, the same scientist who performed drug treatments (where applicable) performed data analysis and proper labeling of each condition was needed to analyze the data.

Reporting for specific materials, systems and methods

We require information from authors about some types of materials, experimental systems and methods used in many studies. Here, indicate whether each material, system or method listed is relevant to your study. If you are not sure if a list item applies to your research, read the appropriate section before selecting a response.

Materials & experimental systems

n/a	Involved in the study
☐	☒ Antibodies
☒	☐ Eukaryotic cell lines
☒	☐ Palaeontology and archaeology
☐	☒ Animals and other organisms
☒	☐ Clinical data
☒	☐ Dual use research of concern

Methods

n/a	Involved in the study
☒	☐ ChIP-seq
☒	☐ Flow cytometry
☒	☐ MRI-based neuroimaging

Antibodies

Antibodies used	primary antibody anti-pcna (Sigma-Aldrich) or anti-BrdU (Sigma-Aldrich), secondary antibody used was Alexa fluor anti-mouse 635
Validation	PCNA and the secondary antibody has been previously validated in Mumm lab studies (see "Immunomodulation-accelerated neuronal regeneration following selective rod photoreceptor cell ablation in the zebrafish retina" David White et. al in 2017 in PNAS). Use of the brdu antibody has been validated in prior zebrafish publications (for example: "A Structural Atlas of the Developing Zebrafish Telencephalon Based on Spatially-Restricted Transgene Expression." K.J Turner et. al in 2022 in Frontiers in Neuroanatomy)

Animals and other research organisms

Policy information about studies involving animals; ARRIVE guidelines recommended for reporting animal research, and Sex and Gender in Research

Laboratory animals	Larval zebrafish from ages 4-11 days old were utilized throughout the study
Wild animals	Study did not involve wild animals
Reporting on sex	Irrelevant to this study, at this stage the larval zebrafish has not determined sex yet
Field-collected samples	Study did not involve samples collected from the field
Ethics oversight	All studies were carried out in accordance with recommendations by the Office of Laboratory Animal Welfare (OLAW) for zebrafish studies and an approved Johns Hopkins University Animal Care and Use Committee animal protocol

Note that full information on the approval of the study protocol must also be provided in the manuscript.
